# Palm vitamin E reduces catecholamines, xanthine oxidase activity and gastric lesions in rats exposed to water-immersion restraint stress

**DOI:** 10.1186/1471-230X-12-54

**Published:** 2012-05-28

**Authors:** Nur Azlina Mohd Fahami, Ibrahim AbdelAziz Ibrahim, Yusof Kamisah, Nafeeza Mohd Ismail

**Affiliations:** 1 Department of Pharmacology, Faculty of Medicine, UKMMC, Universiti Kebangsaan Malaysia, Kuala Lumpur, Malaysia; 2 Department of Pharmacology, Faculty of Medicine, Universiti Teknologi MARA, Shah Alam, Selangor, Malaysia

## Abstract

**Background:**

This study examined the effects of Palm vitamin E (PVE) and α-tocopherol (α-TF) supplementations on adrenalin, noradrenalin, xanthine oxidase plus dehydrogenase (XO + XD) activities and gastric lesions in rats exposed to water-immersion restraint stress (WIRS).

**Methods:**

Sixty male *Sprague–Dawley* rats (200-250 g) were randomly divided into three equal sized groups. The control group was given a normal diet, while the treated groups received the same diet with oral supplementation of PVE or α-TF at 60 mg/kg body weight. After the treatment period of 28 days, each group was further subdivided into two groups with 10 rats without exposing them to stress and the other 10 rats were subjected to WIRS for 3.5 hours. Blood samples were taken to measure the adrenalin and noradrenalin levels. The rats were then sacrificed following which the stomach was excised and opened along the greater curvature and examined for lesions and XO + XD activities.

**Results:**

The rats exposed to WIRS had lesions in their stomach mucosa. Our findings showed that dietary supplementations of PVE and α-TF were able to reduce gastric lesions significantly in comparison to the stressed control group. WIRS increased plasma adrenalin and noradrenalin significantly. PVE and α-TF treatments reduced these parameters significantly compared to the stressed control.

**Conclusions:**

Supplementations with either PVE or α-TF reduce the formation of gastric lesions. Their protective effect was related to their abilities to inhibit stress induced elevation of adrenalin and noradrenalin levels as well as through reduction in xanthine oxidase and dehydrogenase activities.

## Background

Stress affects psychological and physiological balances which can lead to various pathological changes. One known pathological stress-induced condition is the formation of gastric lesions and studies had shown that its pathogenesis is multifactorial. It includes factors which disrupt the gastric mucosal integrity such as changes in gastric acid, mucus and bicarbonate secretions, inhibition of gastric mucosal prostaglandin synthesis [1], reduction of gastric mucosal blood flow [2,3] as well as changes in stress hormones [4-6] and gastric motility [7,8]. It is also known that an increase in catecholamine levels during stress causes vasoconstriction [6]. These changes can ultimately result in formation of gastric lesions.

Recent studies had also shown the involvement of oxidative stress in the pathogenesis of stress-induced gastric ulcer [9,10]. One particular type of oxidant injury is reoxygenation injury following reperfusion of ischemic tissues [11]. Xanthine oxidoreductase exists in two interconvertible forms, which are xanthine dehydrogenase and the oxygen-dependent xanthine oxidase. In some studies, it was shown that allopurinol reduced gastrointestinal injury, in rats that were exposed to xanthine/hypoxanthine + xanthine oxidase system [12,13].

There have been some previous literatures related to the role and ability of vitamin E or its derivatives to reduce stress and gastric lesions. Our previous studies had found that both tocopherol and tocotrienol had the ability to reduce the formation of gastric lesions induced by stress in rats [7,14]. Although tocopherol is well known to be the most available and active form of vitamin E, recently the role of tocotrienols has received renewed attention. The present study was designed to compare the effects of palm vitamin E which mainly contains tocotrienols and α-tocopherol supplementation on catecholamines and gastric xanthine oxidase activity, which are involved in stress-induced gastric lesions in rats.

## Methods

Sixty male *Sprague–Dawley* rats (200–250 gram) were divided into three equal sized groups. The first and second groups were given palm vitamin E (PVE) or α-tocopherol (α-TF) respectively at the dose of 60 mg/kg body weight orally for 28 days, while the control group was given olive oil by using a 4-inch, 18 G needle, as the vehicle. Palm vitamin E used in this study contained a mixture of 22% tocopherol and 78% tocotrienols which was obtained from Malaysia Palm Oil Board (MPOB). The vitamin E dose was chosen based on our previous study, which showed the ability of this dose to reduce gastric lesions occurrence [6]. At the end of treatment period, blood was withdrawn and each group was subdivided into another two groups; one group was subjected to WIRS for 3.5 hours and the other group was not subjected to any stress (non-stress group). The rats were deprived of food overnight before they were exposed to stress.

Stress was conducted by placing each rat in a plastic restrainer individually, after which they were immersed neck-deep in a beaker at room temperature (23°C) for 3.5 hours. This procedure was done following the method by Nishida et al. (1997) [15]. After exposure to stress, the rats were anesthetized by injecting both ketamine (5 mg/100 g body weight) and xylazine (1 mg/100 g body weight) before blood was withdrawn for catecholamine level determination. The rats were then sacrificed after which the stomach was removed. The experimental design was approved by Universiti Kebangsaan Malaysia Animal Ethics Committee (UKMAEC).

### Assessment of gastric lesions

Gastric lesions were measured under 3X magnification using light microscopy. Lesion size in mm was determined by measuring each lesion along its greatest diameter. Each five petechial lesions was equal to 1 mm lesion. The total lengths in each group of rats were averaged and expressed as the lesion index. This method was previously described by Wong et al. [16].

### Gastric xanthine oxidase and xanthine dehydrogenase activities

Tissue preparation for the measurement of xanthine oxidase and xanthine dehydrogenase was done following a method previously described by Qu et al. [17]. The measurement of xanthine oxidase and xanthine dehydrogenase activities followed the method described by Terao et al. [18].

### Plasma adrenalin and noradrenalin

Plasma adrenalin (epinephrine) and noradrenalin (norepinephrine) level were measured using Enzyme Immuno Assay (EIA) kits from IBL-Hamburg, Germany (Catalog number 40-371-25001).

### Statistical analysis

Statistical analysis was carried out using the SPSS statistical package version 12 (SPSS Inc. USA). Normal distribution of all variables was examined by Kolmogorov-Smirnov test. The results were expressed as the means ±standard errors of the mean (SEM). Statistical significance (P < 0.05) was determined by ANOVA followed by Tukey’s post-hoc test.

## Results

### Effects of PVE and α-TF on gastric lesions

**Figure 1:**
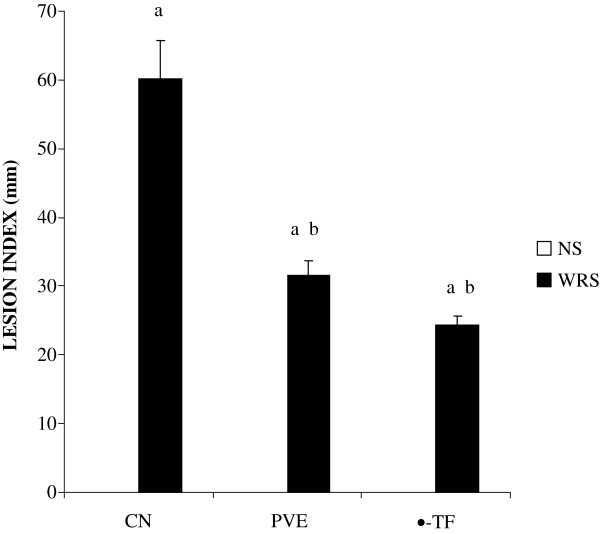
**The gastric lesions number (in millimetres) of rats that were pretreated with palm vitamin E (PVE) or α-tocopherol (α-TF) for 28 days and exposed to water-immersion restraint stress for 3.5 hours.** Bars represent means ± sem (n = 7). a; significantly different from the non-stressed group (CN + NS), b; significantly different from the stressed control (CN + WIRS)(ANOVA followed by Tukeys test, p < 0.05).

**Figure 2:**
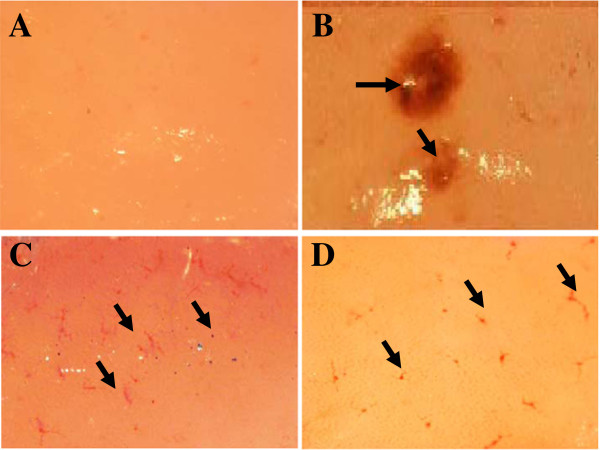
**Microscopic observations (3X) of water-immersion restraint stress (WIRS) induced gastric lesions. A**: Gastric tissue of normal rat (no lesions). **B**: Gastric tissue of a rat exposed to 3.5 h of WIRS (Developed gastric ulcer as shown by the arrow). **C**: Gastric tissue of a rat exposed to 3.5 h of WIRS plus palm vitamin E (PVE) (Developed petichae hemorrhage as shown by the arrows). **D**: Gastric tissue of a rat exposed to 3.5 h of WIRS plus α-tocopherol (α-TF) (Developed petichial hemorrhage as shown by the arrows).

Non-stressed rats showed no focal lesions in the gastric mucosa. However, gastric mucosal lesions developed in rats subjected to water-immersion restraint stress (WIRS) for 3.5 hours. The area of involvement was confined to the glandular part of the stomach. In rats exposed to stress, pretreatments with either palm vitamin E (PVE) or α-tocopherol (α-TF) significantly reduced number of gastric lesions, by 52% (P = 0.001) and 40% (P = 0.001) respectively (Figure 1). Macroscopic observation showed either lesions, most often 1–2 mm in size, or petechial bleeding (Figure 2).

### Effects of PVE and α-TF on noradrenalin

**Figure 3:**
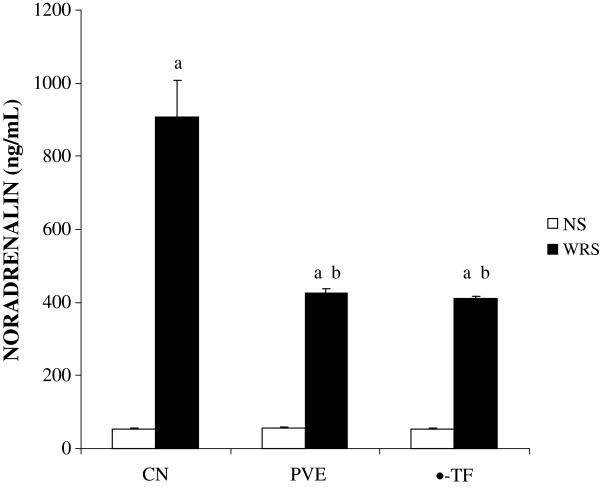
**The plasma noradrenalin level in rats that were pretreated with palm vitamin E (PVE) or α-tocopherol (α-TF) for 28 days and exposed to water-immersion restraint stress for 3.5 hours.** Bars represent means ± sem (n = 7). a; significantly different from the non-stressed group (CN + NS), b; significantly different from the stressed control (CN + WIRS)(ANOVA followed by Tukeys test, p < 0.05).

Figure 3 shows that the exposure to WIRS for 3.5 hours increased the plasma noradrenalin level significantly (about 92%, P =0.001). The plasma noradrenalin levels of stressed PVE- (about 59%, P = 0.025) and α-TF-treated groups (about 70%, P =0.022) were decreased significantly compared to the stressed control group. However, no significant difference was observed in the plasma noradrenalin level between the stressed PVE and α-TF groups. The exposure to WIRS for 3.5 hours increased plasma noradrenalin level significantly in PVE- (P = 0.001) and α-TF-treated groups (P = 0.001) in comparison to their respective non-stressed groups. No significant difference (P > 0.05) in the plasma noradrenalin level between the non-stressed groups was observed.

### Effects of PVE and α-TF on adrenalin

**Figure 4:**
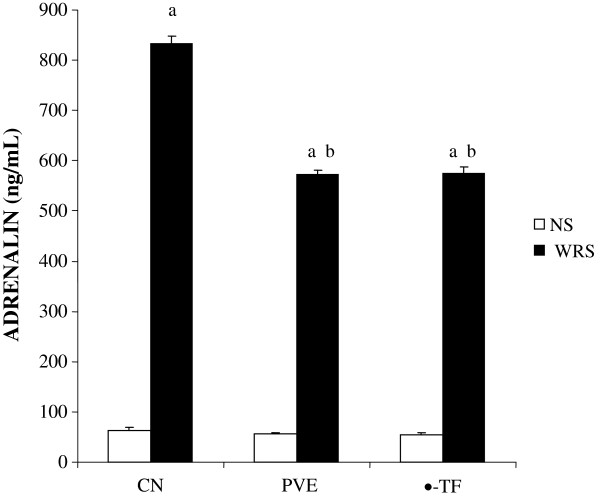
**The plasma adrenalin level in rats that were pretreated with palm vitamin E (PVE) or α-tocopherol (α-TF) for 28 days and exposed to water-immersion restraint stress for 3.5 hours.** Bars represent means ± sem (n = 7). a; significantly different from the non-stressed group (CN + NS), b; significantly different from the stressed control (CN + WIRS)(ANOVA followed by Tukeys test, p < 0.05).

The output presented in Figure 4 shows that immobilization stress increased the adrenalin level significantly compared to non-stressed group (about 89%, P =0.003). There was a significant reduction in the adrenalin level of stressed PVE- (about 18.7%, P = 0.002) and α-TF-treated groups (about 20%, P =0.001) compared to the stressed controls. However, no significant difference in the adrenalin level between stressed PVE- and α-TF-treated groups was seen. In addition, the exposure to WIRS increased plasma adrenalin level significantly in PVE- and α-TF-treated groups compared to their respective non-stressed groups. No significant difference in the adrenalin levels between the non-stressed groups was observed.

### Effects of PVE and α-TF on (XO + XD) activity

**Figure 5:**
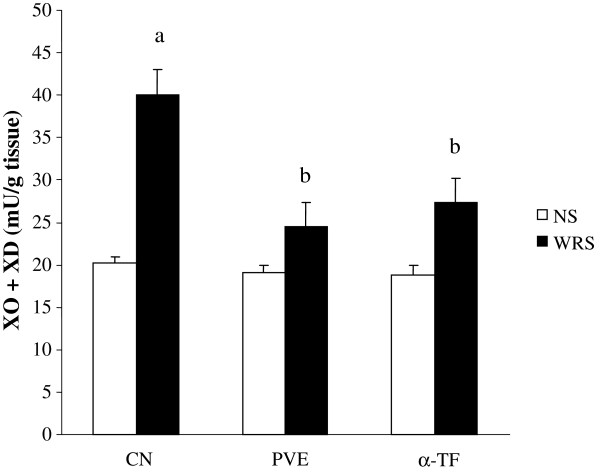
**The gastric xanthine oxidase + xanthine dehydrogenase (XO + XD) activity in the stomach of rats that were pretreated with palm vitamin E (PVE) or α-tocopherol (α-TF) for 28 days and exposed to water-immersion restraint stress for 3.5 hours.** Bars represent means ± sem (n = 7). a; significantly different from the non-stressed group (CN + NS), b; significantly different from the stressed control (CN + WIRS)(ANOVA followed by Tukeys test, p < 0.05).

Figure 5 shows the activities of xanthine oxidase plus xanthine dehydrogenase (XO + XD). The water-immersion restraint stress (WIRS) significantly increased the activities of XO + XD by 76% (P = 0.003) compared to the non-stressed control. The activities of XO + XD of stressed PVE- and α-TF-treated groups were reduced significantly compared to the stressed control. However, there was no significant difference in the activities of XO + XD between the stressed PVE- and α-TF-treated groups. In addition, no significant differences in the activities of XO + XD were seen in the PVE and α-TF stressed group compared to their respective non-stressed group.

## Discussion

The increase in the noradrenalin and adrenalin levels due to stress are well documented [19-21]. The present study showed that exposure to water-immersion restraint stress (WIRS) for 3.5 hours was enough to increase the level of these catecholamines significantly; noradrenalin by 92% and adrenalin by 89%. These observations support the hypothesis that adrenal catecholamines play a physiological role in response to stressful situations. Hamada et al. found that rats exposed to stress developed gastric lesions associated with reduced brain noradrenalin content and increased plasma catecholamines and corticosterone levels [22]. Similarly, we had previously shown that rats exposed to repeated restraint stress had a higher level of plasma noradrenalin and corticosterone compared to the non-stressed rats [6].

During stress, the underlying mechanisms involved are the activation of the hypothalamic-pituitary-adrenal axis (HPA) and sympatho-adrenal-medullary (SAM) systems, causing the release of corticosterone together with the release of noradrenalin and adrenalin [23]. Furthermore, the elevation in catecholamine levels may generate free radicals [24], which may be cytotoxic and mediate tissue damage by injuring cellular membranes and releasing intracellular components. It is widely accepted that the pathogenesis of gastric mucosal lesions involves oxygen-derived free radicals.

In the present study, the noradrenalin and adrenalin levels of stressed PVE and α-TF groups were reduced significantly in comparison to the stressed control. In parallel to its ability to block noradrenalin, vitamin E also blocked formation of gastric lesions in the rats exposed to stress. Moreover, the noradrenalin and adrenalin levels in the stressed PVE- and α-TF-treated groups were not different from their respective non-stressed groups. This suggests that vitamin E plays an important role in reducing the elevated catecholamine levels induced by stress. We had previously reported that the increase in the noradrenalin level was blocked in rats given tocotrienols supplementation but not in rats receiving α-TF [6]. These findings suggest that tocotrienols are more potent than α-TF in blocking the effects of stress. However, we found no significant difference between the stressed PVE- and α-TF-treated groups. Both treatments were able to improve the effects of stress by reducing the levels of noradrenalin and adrenalin. The differences observed could be due to the different stress models used; acute versus repeated stress. In 2007, Campese and Shaohua showed that rats fed with a vitamin-E-fortified diet manifested a significant reduction in noradrenalin secretion from the posterior hypothalamus [25]. A vitamin-E-fortified diet mitigated the formation of reactive oxygen species in the brain, and this was associated with a reduced sympathetic nervous system activity and blood pressure in rats with phenol-induced renal injury.

Lipid peroxidation mediated by free radicals is considered a primary mechanism of cell membrane destruction [26]. Gastric lesions caused by stress, alcohol, *Helicobacter pylori* infection and non-steroidal anti-inflammatory drugs have been shown to be mediated largely through the generation of reactive oxygen species (ROS) that seems to play an important role in producing lipid peroxides [3,14,27,28]. The damage in gastric mucosa due to WIRS has been attributed to impaired gastric microcirculation, which results in ischemia followed by reperfusion, a process that generates free radicals. The finding indicates that reactive oxygen species and lipid peroxidation are important in the pathogenesis of gastric mucosal injury induced by stress [10]. This present finding is consistent with the elevation of XO activity after stress, which produces ROS. A previous study had indicated that the exposure of rats to 3.5 hours of WIRS led to an increase in the xanthine metabolism to the level comparable to that observed in ischaemia-reperfusion model of gastric injury [2]. Xanthine oxidase activity is a major source of ROS such as superoxide anion (O_2_^·–^) and hydrogen peroxide (H_2_O_2_) in the pathogenesis of disease in various biological systems including gastrointestinal tract [29-31]. The increase in ROS would then increase the gastric lipid peroxidation and subsequent gastric lesion development. This supports the hypothesis that stress-induced injury is mediated by lipid peroxidation.

In the present study, PVE and α-TF had prevented the increase in XO + XD activities significantly after WIRS. It could be that both PVE and α-TF improved the gastric mucosal blood flow that was impaired during WIRS [2,32]. Improved gastric blood flow would further suppress the conversion of XD to XO. Raghuvanshi et al. showed that administration of 400 mg of vitamin E for six days along with 80 mg of aspirin produced an excellent antioxidant effect as evidenced by a reduced platelet xanthine oxidase activity [33].

Vitamin E is a lipid-soluble antioxidant and a well accepted first line defence mechanism against lipid peroxidation. It functions as a chain-breaking antioxidant for lipid peroxidation in cell membranes and as a scavenger of ROS such as superoxide anion, hydrogen peroxide and singlet oxygen [34]. Yoshikawa et al. reported a decrease in gastric mucosal vitamin E level and an increase in gastric mucosal lipid peroxidation in ischemia-reperfusion-induced gastric mucosal injury and the severity of the injury was enhanced in vitamin E-deficient rats [35]. Naito et al. had shown that in nitric oxide-depleted rats, vitamin E played an important protective role against ischemia-reperfusion-induced gastric mucosal injury, and suggested that this gastroprotective effect of vitamin E was not only due to its antioxidant action but also its inhibitory action on neutrophil infiltration into the gastric mucosa [36]. Al-Tuwaijri and Al-Dhohyan reported that a single oral pre-administration of α-tocopherol acetate to rats prevented ischemia-reperfusion-induced gastric mucosal injury [37].

As mentioned earlier, stress can impair gastric blood flow and cause ischemic-like conditions. These conditions can lead to reperfusion-induced injury and finally development of gastric lesions. During ischemia-reperfusion, lipid peroxidation was increased due to the production of ROS; supplementations with PVE and α-TF were able to reduce this increase. It can be concluded that PVE and α-TF have gastroprotective effects against WIRS, possibly via their antioxidant properties. As shown in this study, animals exposed to WIRS for 3.5 hours developed gastric mucosal lesions, thus confirming the reproducibility of this model for the study. Supplementations of PVE and α-TF at 60 mg/kg for 28 days prior to exposure to stress reduced the gastric mucosal injury. However, no difference between these two agents was observed, showing equal effectiveness in preventing stress-induced gastric injury.

Similarly, exposure to WIRS has been shown to increase the incidence of gastric mucosal lesion and the increase was lowered by the administration of various antioxidants [1,38]. A study by Ohta et al. had demonstrated that WIRS for 6 hours reduced gastric α-tocopherol concentration but pre-administration of ascorbic acid partially reversed this reduction. In the present study, the prevention of the harmful effects of stress on the gastric mucosa may be mediated by the antioxidant activity possessed by PVE and α-TF, which reduce the formation of free radicals either directly or indirectly, leading to attenuation of lesion formation.

The protective mechanism of vitamin E and its role on human health is still not well understood. The antioxidant characteristic of vitamin E, especially its effect on polyunsaturated fatty acids (PUFA) may improve cell membrane integrity. There is possibility that the gastric tissues become more resistant towards the aggressive factors like acid and pepsin.

## Conclusions

Our data suggest that the protective effect of vitamin E was related to a decreased xanthine oxidase and dehydrogenase activities, which resulted in a reduction in the formation of free radicals. There is also a possibility that the ability of both PVE and α-tocopherol in blocking the stress induced damages was through its action on a higher level which was by blocking the increased in adrenalin and noradrenalin, known mediators of stress.

## Competing interests

The authors declare that they have no competing interests.

## Authors' contributions

All authors contributed to the study design and interpretation of the data. IAI was responsible for the experimental work and data collection. NMF contributed to the preparation of the manuscript, while other authors (IAI, KY and NMI) had revised the manuscript critically and approved its final version. All authors read and approved the final manuscript.

## Pre-publication history

The pre-publication history for this paper can be accessed here:

http://www.biomedcentral.com/1471-230X/12/54/prepub
